# Supplementary material for: Effects of Acupuncture-Related Therapies in the Rehabilitation of Patients with Post-Stroke Aphasia—A Network Meta-Analysis of Randomized Controlled Trials
Source: Brain Sci. 2022 Sep 23;12(10):1282. doi: 10.3390/brainsci12101282 (PMC9599621; doi:10.3390/brainsci12101282)
Supplement: Supplementary file 1 [file brainsci-12-01282-s001.zip › Supplementary materials.pdf]

Supplementary Table S1. Search strategy (take PubMed as an example)

#1	"Stroke"[Mesh] OR Cerebrovascular Accident[Title/Abstract] OR CVA OR CVAs OR Cerebrovascular Apoplexy[Title/Abstract] OR Brain Vascular Accident[Title/Abstract] OR Vascular Accidents, Brain[Title/Abstract] OR Cerebrovascular Stroke[Title/Abstract] OR Stroke, Cerebrovascular[Title/Abstract] OR Apoplexy[Title/Abstract] OR Cerebral Stroke[Title/Abstract] OR Acute Stroke[Title/Abstract] OR Acute Cerebrovascular Accident[Title/Abstract]
#2	"Aphasia"[Mesh] OR Alogia[Title/Abstract] OR Word Deafness[Title/Abstract] OR Anepia[Title/Abstract] OR Semantic Aphasia[Title/Abstract] OR Syntactical Aphasia[Title/Abstract] OR Progressive Aphasia[Title/Abstract] OR Acquired Aphasia[Title/Abstract] OR Ageusic Aphasia[Title/Abstract] OR Auditory Discriminatory Aphasia[Title/Abstract] OR Commisural Aphasia[Title/Abstract] OR Functional Aphasia[Title/Abstract] OR Global Aphasia[Title/Abstract] OR Graphomotor Aphasia[Title/Abstract] OR Intellectual Aphasia[Title/Abstract] OR Mixed Aphasia[Title/Abstract] OR Post-Ictal Aphasia[Title/Abstract] OR Post-Traumatic Aphasia[Title/Abstract] OR Dejerine Lichtheim Phenomenon[Title/Abstract] OR Lichtheim Sign[Title/Abstract] OR Dysphasia[Title/Abstract]
#3	#1 and #2
#4	Post-stroke Aphasia[Title/Abstract] OR post stroke Aphasia[Title/Abstract] OR Aphasia after stroke[Title/Abstract] OR poststroke Aphasia[Title/Abstract]
#5	#3 or #4
#6	"Acupuncture"[Mesh] OR Auricular Acupuncture[Title/Abstract] OR Ear Acupuncture[Title/Abstract] OR Auricular Acupunctures[Title/Abstract] OR auriculoacupuncture[Title/Abstract] OR earlobe acupuncture[Title/Abstract] OR "Acupuncture Points"[Mesh] OR Acupuncture Point[Title/Abstract] OR "Acupuncture Therapy"[Mesh] OR Acupuncture Treatment[Title/Abstract] OR "acupressure" [Mesh] OR Zhi Ya[Title/Abstract] OR Scalp acupuncture[Title/Abstract] OR warm acupuncture[Title/Abstract] OR burnt needle therapy[Title/Abstract] OR fire acupuncture[Title/Abstract] OR fire needle acupuncture[Title/Abstract] OR heat acupuncture[Title/Abstract] OR thermal acupuncture[Title/Abstract] OR thermoacupuncture[Title/Abstract] OR Electroacupuncture[Title/Abstract] OR electric acupuncture[Title/Abstract] OR electrical acupoint stimulation[Title/Abstract] OR electrical acupuncture[Title/Abstract] OR electro-acupuncture[Title/Abstract] OR electronic acupuncture[Title/Abstract] OR Catgut embedding[Title/Abstract] OR acupoint catgut embedding therapy[Title/Abstract] OR catgut implantation[Title/Abstract] OR Pharmacoacupuncture[Title/Abstract] OR herb acupuncture[Title/Abstract] OR herbalized acupuncture[Title/Abstract] OR pharmaco-acupuncture[Title/Abstract] OR pharmacoacupuncture[Title/Abstract]

	OR point injection[Title/Abstract]
#7	randomized controlled trial[Publication Type] OR randomized[Title/Abstract] OR placebo[Title/Abstract]
#8	#5 and #6 and #7

We also searched three Chinese language databases databases: CNKI, Wanfang Data, Chongqing VIP to retrieve relevant studies. The subject words and synonyms related to this study were identified in Sinomed. To acquire better retrieval results, we kept the search terms as broad as possible to identify the relevant publications, including the following Chinese keywords: "stroke", "aphasia", "cerebrovascular disease", "brain ischemia", "hemorrhage", "ischemic stroke", "cerebral vascular accident", "cerebral infarction", "aphasia", "aphasia", "language barrier", "language barrier", "acupuncture therapy" "Acupuncture", "electric needle", "tongue needle", "head needle", "puncture", "ear needle", "fire needle", "warm needle"，RCT experiments were further screened out from the retrieval results.

I) CNKI

SU%='卒中'+'脑中风'+'脑血管病'+'脑缺血'+'脑出血'+'缺血性脑卒中'+'脑血管意外'+'脑梗' AND SU%='失语'+'失语症'+'言语障碍'+'语言障碍' AND SU%='针灸疗法'+'针刺'+'电针'+'舌针'+'头皮针'+'刺络'+'耳针'+'火针'+'温针'

II) Wanfang Data

主题:(卒中 or 脑中风 or 脑血管病 or 脑缺血 or 脑出血 or 缺血性脑卒中 or 脑血管意外 or 脑梗) and 主题:(失语 or 失语症 or 言语障碍 or 语言障碍) and 主题:(针灸疗法 or 针刺 or 电针 or 舌针 or 头皮针 or 刺络 or 耳针 or 火针 or 温针)

III) Chongqing VIP

题名或关键词=卒中+脑中风+脑血管病+脑缺血+脑出血+缺血性脑卒中+脑血管意外+脑梗 AND 题名或关键词=失语+失语症+言语障碍+语言障碍 AND 题名或关键词=针灸疗法+针刺+电针+舌针+头皮针+刺络+耳针+火针+温针(同义词已扩展)

Supplementary Table S2. Details of the intervention measures

Study	Interventions		Baseline comprehension score	Baseline oral expression score	Baseline repetition score	Baseline denomination score
	T	C				
Bao 2020	SLT+BA+TA<br>Treatment course: 4 weeks<br>Frequency: 5 times a week<br>Duration: 30 min	SLT<br>Treatment course: 4 weeks<br>Frequency:5 times a week<br>Duration: 30 min	-	-	-	-
Chen 2006	SLT+BA+SA+TA<br>Treatment course: 8 weeks<br>Frequency: once a day<br>Duration: 30 min	SLT<br>Treatment course: 8 weeks<br>Frequency: once a day<br>Duration: 30 min	-	-	-	-
Chen X 2021	SLT+BA<br>Treatment course: 8 weeks<br>Frequency: 6 times a week<br>Duration: 40 min	SLT<br>Treatment course: 8weeks<br>Frequency: 6 times a week<br>Duration: 40 min	T: 158.73±4.20<br>C: 158.40±4.10	-	T: 20.20±3.49<br>C: 20.18±3.59	T: 12.74±3.08<br>C: 12.70±3.11
Cheng X 2018	SLT+SA<br>Treatment course: 4 weeks<br>Frequency: twice a day<br>Duration: 60 min	SLT<br>Treatment course: 4 weeks<br>Frequency: twice a day<br>Duration: 30 min	-	T: 7.22±2.37<br>C: 7.16±2.75	T: 39.23±8.52<br>C: 39.94±7.92	T: 23.75±7.50<br>C: 24.84±8.64
Dong X 2020	SLT+SA<br>Treatment course: 2 weeks<br>Frequency: 5 times a week<br>Duration: 30 min	SLT<br>Treatment course: 2 weeks<br>Frequency: 5 times a week<br>Duration: 30 min	-	-	-	-

Supplementary Table S2. *Cont*

Study	Interventions		Baseline comprehension score	Baseline oral expression score	Baseline repetition score	Baseline denomination score
	T	C				
Dong Y 2009	SLT+BA<br>Treatment course: 8 weeks<br>Frequency: once a day<br>Duration: 45 min	SLT<br>Treatment course: 8 weeks<br>Frequency: once a day<br>Duration: 45 min	-	-	-	-
Feng X 2007	SLT+BA+SA<br>Treatment course: one month<br>Frequency: once a day<br>Duration: 30 min	SLT<br>Treatment course: one month<br>Frequency: once a day<br>Duration: 30 min	-	-	-	-
He 2021	SLT+BA<br>Treatment course: 8 weeks<br>Frequency: 6 times a week<br>Duration: 40 min	SLT<br>Treatment course: 8weeks<br>Frequency: 6 times a week<br>Duration: 40 min	-	-	-	-
He A 2014	SLT+TA<br>Treatment course: 20 days<br>Frequency: once a day<br>Duration: 30 min	SLT<br>Treatment course: 20 days<br>Frequency: NA<br>Duration: NA	-	-	-	-
Hou 2018	SLT+BA+SA<br>Treatment course: 4 weeks<br>Frequency: twice a day<br>Duration: 30 min	SLT<br>Treatment course: 4 weeks<br>Frequency: NA<br>Duration: NA	-	-	-	-

Supplementary Table S2. *Cont*

Study	Interventions		Baseline comprehension score	Baseline oral expression score	Baseline repetition score	Baseline denomination score
	T	C				
Hou W 2012	BA<br>Treatment course: 2 weeks<br>Frequency: once a day<br>Duration: 40 min	SLT<br>Treatment course: 2 weeks<br>Frequency: twice a day<br>Duration: 20 min	T: 110.08±30.13<br>C: 113.00±29.78	T: 6.32±3.74<br>C: 6.47±4.38	T: 47.88±17.52<br>C: 48.12±14.64	T: 45.14±16.79<br>C: 45.76±21.31
Huang H 2009	SLT+SA<br>Treatment course: one month<br>Frequency: once a day<br>Duration: 40 min	SLT<br>Treatment course: one month<br>Frequency: once a day<br>Duration: 40 min	-	-	-	-
Huang S 2016	SLT+BA<br>Treatment course: one month<br>Frequency: once a day<br>Duration: 30 min	BA<br>Treatment course: one month<br>Frequency: once a day<br>Duration: 30 min	-	-	-	-
Jiang 2008	BA+TA<br>Treatment course: 4 weeks<br>Frequency: 5 times a week<br>Duration: 30 min	BA<br>Treatment course: 4 weeks<br>Frequency: 5 times a week<br>Duration: 30 min	T: 134.75±10.96<br>C: 131.53±10.50	T: 18.98±2.49<br>C: 19.09±2.72	T: 38.63±3.01<br>C: 39.12±2.62	T: 43.10±6.71<br>C: 36.29±7.07
Li 2019	TA<br>Treatment course: 20 days<br>Frequency: 6 times a week<br>Duration: 30 min	BA<br>Treatment course: 20 days<br>Frequency: 6 times aweek<br>Duration: 30 min	-	T: 11.06±3.65<br>C: 10.89±3.38	T: 44.56±8.45<br>C: 45.16±8.79	-

Supplementary Table S2. *Cont*

Study	Interventions T	Interventions C	Baseline comprehension score	Baseline oral expression score	Baseline repetition score	Baseline denomination score
Li L 2020	BA+TA<br>Treatment course: 4 weeks<br>Frequency: five times a week<br>Duration: NA	BA<br>Treatment course: 4 weeks<br>Frequency: five times a week<br>Duration: NA	T: 169.28±8.75<br>C: 173.27±5.53	T: 17.79±3.76<br>C: 19.09±3.22	T: 25.58±3.25<br>C: 26.42±3.49	T: 17.33±2.91<br>C: 17.15±2.12
Li G 2006	SLT+BA<br>Treatment course: NA<br>Frequency: once a day<br>Duration: 50-90 min	SLT<br>Treatment course: NA<br>Frequency: once a day<br>Duration: 30-60 min	-	-	-	-
Li Q 2017	SLT+BA+SA<br>Treatment course: one month<br>Frequency: once a day<br>Duration: 35-40 min	SLT<br>Treatment course: one month<br>Frequency: once a day<br>Duration: 30 min	-	-	-	-
Li S 2014	SLT+SA+TA<br>Treatment course: 3 months<br>Frequency: 3 times a week<br>Duration: 60 min	SLT<br>Treatment course: 3 months<br>Frequency: 3 times a week<br>Duration: 60 min	-	-	-	-
Li X 2009	BA+TA<br>Treatment course: 4 weeks<br>Frequency: 5 times a week<br>Duration: 30 min	BA<br>Treatment course: 4 weeks<br>Frequency: 5 times a week<br>Duration: 30 min	T: 174.75±10.96<br>C: 171.53±10.50	T: 10.08±1.89<br>C: 9.82±2.72	T: 28.63±3.01<br>C: 29.12±2.62	T: 18.12±2.77<br>C: 17.66±2.86

Supplementary Table S2. *Cont*

Study	Interventions		Baseline comprehension score	Baseline oral expression score	Baseline repetition score	Baseline denomination score
	T	C				
Li Y 2019	SLT+BA+SA<br>Treatment course: 3 months<br>Frequency: once a day<br>Duration: 30 min	SLT<br>Treatment course: 3 months<br>Frequency: once a day<br>Duration: 30 min	-	-	-	-
Li Z 2019	SLT+BA+TA<br>Treatment course: 3 months<br>Frequency: 6 times a day<br>Duration: 30min (BA+TA)	SLT<br>Treatment course: 3 months<br>Frequency: 6 times a day<br>Duration: NA	T: 161.31±7.74<br>C: 163.20±7.70	T: 19.64±2.64<br>C: 17.34±2.15	T: 26.65±3.23<br>C: 24.77±2.34	T: 16.76±2.72<br>C: 18.71±3.68
Li ZZ 2019	SLT+TA<br>Treatment course: 3 weeks<br>Frequency: once a day<br>Duration: 30 min	SLT<br>Treatment course: one month<br>Frequency: once a day<br>Duration: 30 min	-	-	-	-
Li ZP 2004	TA<br>Treatment course: 24 days<br>Frequency: once a day<br>Duration: NA	BA<br>Treatment course: 24 days<br>Frequency: once a day<br>Duration: NA	-	-	-	-
Li ZP 2005	TA<br>Treatment course: 24 days<br>Frequency: once a day<br>Duration: NA	BA<br>Treatment course: 24 days<br>Frequency: once a day<br>Duration: NA	-	-	-	-

Supplementary Table S2. *Cont*

Study	Interventions		Baseline comprehension score	Baseline oral expression score	Baseline repetition score	Baseline denomination score
	T	C				
Liu 2018	BA<br>Treatment course: 12 weeks<br>Frequency: 5 times a week<br>Duration: 50 min	SLT<br>Treatment course: 12 weeks<br>Frequency: 6 times a week<br>Duration: 45 min	-	-	-	-
Liu 2018	BA<br>Treatment course: 12 weeks<br>Frequency: 5 times a week<br>Duration: 20 min	SLT<br>Treatment course: 12 weeks<br>Frequency: 5 times a week<br>Duration: 30 min	-	-	-	-
Liu J 2018	BA<br>Treatment course: 14 weeks<br>Frequency: 5 times a week<br>Duration: 20 min	SLT<br>Treatment course: 14 weeks<br>Frequency: 5 times a week<br>Duration: 40 min	T: 97.75±16.15<br>C: 96.10±15.46	T: 19.42±5.75<br>C: 19.09±6.64	T: 28.65±8.01<br>C: 28.88±8.43	T: 29.11±8.84<br>C: 31.39±9.10
Liu L 2006	SLT+BA<br>Treatment course: one month<br>Frequency: once a day<br>Duration: 60 min	SLT<br>Treatment course: one month<br>Frequency: once a day<br>Duration: 30 min				
Liu X 2011	TA<br>Treatment course: 20 days<br>Frequency: once a day<br>Duration: NA	BA<br>Treatment course: 20 days<br>Frequency: once a day<br>Duration: NA				

Supplementary Table S2. *Cont*

Study	Interventions		Baseline comprehension score	Baseline oral expression score	Baseline repetition score	Baseline denomination score
	T	C				
Lu Q 2010	SLT+BA+SA<br>Treatment course: one month<br>Frequency: once a day<br>Duration: 60 min	SLT<br>Treatment course: one month<br>Frequency: once a day<br>Duration: 30 min	T: 14.25±11.43<br>C: 18.00±12.09	T: 14.25±11.40<br>C: 12.75±9.44	-	-
Luo 2008	SLT+TA<br>Treatment course: one month<br>Frequency: once a day<br>Duration: NA	SLT<br>Treatment course: one month<br>Frequency: 5-10 times aday<br>Duration: 5-10 min	T: 12.38±5.76<br>C: 13.13±6.06	T: 14.28±5.77<br>C: 15.76±5.34	T: 11.51±4.55<br>C: 12.64±5.29	T: 6.74±2.36<br>C: 6.91±3.59
Luo 2008	SLT+BA<br>Treatment course: one month<br>Frequency: once a day<br>Duration: 30 min	SLT<br>Treatment course: one month<br>Frequency: 5-10 times a day<br>Duration: 5-10 min	T: 12.38±5.77<br>C: 13.13±6.06	T: 14.28±5.76<br>C: 15.76±5.34	T: 11.51±4.55<br>C: 12.64±5.29	T: 6.74±2.36<br>C: 6.91±3.59
Mi 2004	TA<br>Treatment course: one month<br>Frequency: once a day<br>Duration: 30 min	BA<br>Treatment course: one month<br>Frequency: once a day<br>Duration: 30 min	-	-	-	-
Qin 2018	SLT+BA<br>Treatment course: 6 weeks<br>Frequency: twice a day<br>Duration: NA	SLT<br>Treatment course: 6 weeks<br>Frequency: twice a day<br>Duration: 30 min	T: 171.45±7.33<br>C: 171.58±7.27	-	T: 23.93±1.59<br>C: 24.08±1.39	T: 15.31±1.36<br>C: 15.25±1.28

Supplementary Table S2. *Cont*

Study	Interventions		Baseline comprehension score	Baseline oral expression score	Baseline repetition score	Baseline denomination score
	T	C				
Qiu 2020	SLT+SA<br>Treatment course: 8 weeks<br>Frequency: 5 times a week<br>Duration: 45 min	SLT<br>Treatment course: 8 weeks<br>Frequency: 5 times a week<br>Duration: 30 min	-	-	-	-
Shi 2021	SLT+BA<br>Treatment course: 3 months<br>Frequency: once a day<br>Duration: 30 min	SLT<br>Treatment course: 3 months<br>Frequency: once a day<br>Duration: 30 min	-	-	-	-
Song 2017	SLT+TA<br>Treatment course: 3 weeks<br>Frequency: 5 times a week<br>Duration: 60 min	SLT<br>Treatment course: 3 weeks<br>Frequency: 5 times a week<br>Duration: 30 min	-	-	-	-
Song Z 2019	SLT+SA<br>Treatment course: 16 weeks<br>Frequency: 4 times a week<br>Duration: 40 min	SLT<br>Treatment course: 16 weeks<br>Frequency: 4 times a week<br>Duration: 40 min	T: 96.31±14.56<br>C: 95.11±15.56	T: 19.34±6.66<br>C: 19.11±6.66	T: 29.21±8.46<br>C: 29.01±8.45	T: 31.49±9.17<br>C: 31.40±9.11
Tian 2012	SLT+BA<br>Treatment course: one month<br>Frequency: once a day<br>Duration: NA	SLT<br>Treatment course: one month<br>Frequency: once a day<br>Duration: NA	-	-	-	-

Supplementary Table S2. *Cont*

Study	Interventions		Baseline comprehension score	Baseline oral expression score	Baseline repetition score	Baseline denomination score
	T	C				
Tong 2017	SLT+SA+TA<br>Treatment course: 4 weeks<br>Frequency: 5 times a week<br>Duration: 90 min	SLT<br>Treatment course: 4 weeks<br>Frequency: 5 times a week<br>Duration: 60 min	T: 158.94±65.78<br>C: 160.21±67.44	-	T: 24.16±11.03<br>C: 23.97±10.42	T: 18.16±5.33<br>C: 17.94±4.92
Wang 2015	SLT+BA+SA<br>Treatment course: 2 months<br>Frequency: 6 times a week<br>Duration:30min (BA+SA)	SLT<br>Treatment course: 2 months<br>Frequency: NA<br>Duration: NA	T: 135.60±16.70<br>C: 135.45±17.08	T: 12.58±3.66<br>C: 11.95±3.55	T: 18.90±4.93<br>C: 18.60±5.97	T: 15.43±3.57<br>C: 15.50±3.58
Wang L 2011	SLT+BA+SA+TA<br>Treatment course: 3 weeks<br>Frequency: 5 times a week<br>Duration: 90 min	SLT<br>Treatment course: 3 weeks<br>Frequency: 5 times a weeek<br>Duration: 60 min	T: 6.95±1.09<br>C: 7.15±1.37	T: 14.03±2.67<br>C: 13.85±2.97	T: 77.03±6.70<br>C: 76.03±6.31	T: 7.33±1.23<br>C: 7.35±1.23
Wang M 2016	SLT+SA+TA<br>Treatment course: 4 weeks<br>Frequency: once a day<br>Duration: 30 min	SLT<br>Treatment course: 4 weeeks<br>Frequency: once a day<br>Duration: 30 min	-	-	-	-
Wang P 1999	TA<br>Treatment course: 10 days<br>Frequency: once a day<br>Duration: 30 min	BA<br>Treatment course: 10 days<br>Frequency: once a day<br>Duration: 30 min	-	-	-	-

Supplementary Table S2. *Cont*

Study	Interventions		Baseline comprehension score	Baseline oral expression score	Baseline repetition score	Baseline denomination score
	T	C				
Wang Q 2018	SLT+TA<br>Treatment course: 6 weeks<br>Frequency: 3 times a week<br>Duration: 30 min	SLT<br>Treatment course: 6weeks<br>Frequency: 3 times a week<br>Duration: 30 min	T: 12.69±2.69<br>C: 12.34±2.73	T: 3.45±1.22<br>C: 3.21±1.10	T: 6.86±1.97<br>C: 6.50±1.95	T: 15.46±4.40<br>C: 15.00±3.92
Wang S 2006	BA<br>Treatment course: 4 weeks<br>Frequency: 5 tiimes a week<br>Duration: 30 min	SLT<br>Treatment course: 4 weeks<br>Frequency: 5 times a week<br>Duration: 30 min	-	-	-	-
Wang T 2016	SLT+TA<br>Treatment course: 4 weeks<br>Frequency: 5 times a week<br>Duration: 50 min	SLT<br>Treatment course: 4 weeks<br>Frequency: 5 times a week<br>Duration: 30 min	-	-	-	-
Wang W 2009	SLT+SA<br>Treatment course: 3 months<br>Frequency: 6 times a week<br>Duration: 30 min	SA<br>Treatment course: 3 months<br>Frequency: 6 times a week<br>Duration: 30 min	T: 34.55±3.36<br>C: 12.34±2.73	T: 3.59±1.14<br>C: 3.61±1.16	-	-
Wang Y 2021	SLT+SA<br>Treatment course: 48 days<br>Frequency: 6 times a week<br>Duration: 60 min	SLT<br>Treatment course: 48 days<br>Frequency: 6 times a week<br>Duration: 30 min	-	-	-	-

Supplementary Table S2. *Cont*

Study	Interventions		Baseline comprehension score	Baseline oral expression score	Baseline repetition score	Baseline denomination score
	T	C				
Wei 2021	SLT+BA<br>Treatment course: 8 weeks<br>Frequency: once a day<br>Duration: 30 min	SLT<br>Treatment course: 8 weeks<br>Frequency: once a day<br>Duration: 30 min	T: 164.44±9.17<br>C: 167.05±9.34	T: 17.56±1.69<br>C: 17.38±1.84	T: 25.12±2.81<br>C: 25.58±2.93	T: 37.02±3.98<br>C: 36.48±4.62
Wei T 2005	SLT+BA+SA+TA<br>Treatment course: NA<br>Frequency: NA<br>Duration: 20-30 min	SLT<br>Treatment course: NA<br>Frequency: NA<br>Duration: 20-30 min	-	-	-	-
Wen 2019	SLT+SA+TA<br>Treatment course: one month<br>Frequency: once a day<br>Duration: 60 min	SLT<br>Treatment course: one month<br>Frequency: once a day<br>Duration: 30 min	T: 143.20±9.25<br>C: 139.82±9.06	T: 32.04±2.02<br>C: 31.04±2.02	T: 60.14±3.06<br>C: 60.13±2.93	T: 40.13±2.01<br>C: 40.02±2.01
Wu 2011	BA<br>Treatment course: 4 weeks<br>Frequency: 5 times a week<br>Duration: 30 min	SLT<br>Treatment course: 4 weeks<br>Frequency: 5 times a week<br>Duration: 30 min	-	-	-	-
Wu M 2008	BA+TA<br>Treatment course: one month<br>Frequency: once a day<br>Duration: 30 min	SLT<br>Treatment course: one month<br>Frequency: once a day<br>Duration: 30 min	-	-	-	-

Supplementary Table S2. *Cont*

Study	Interventions T	Interventions C	Baseline comprehension score	Baseline oral expression score	Baseline repetition score	Baseline denomination score
Xie M 2015	SLT+BA+TA<br>Treatment course: 45 days<br>Frequency: once a day<br>Duration: 30 min (BA+TA)	SLT<br>Treatment course: 45 days<br>Frequency: once a day<br>Duration: NA	-	-	-	-
Xu 2021	BA+SA<br>Treatment course: 4 weeks<br>Frequency: 5 times a day<br>Duration: 30 min	SA<br>Treatment course: 4 weeks<br>Frequency: 5 times a day<br>Duration: 30 min	T: 55.51±7.87<br>C: 55.49±7.84	T: 57.36±6.21<br>C: 57.41±6.45	T: 59.56±7.25<br>C: 59.49±7.27	T: 56.48±7.67<br>C: 56.37±7.62
Xu M 2021	SLT+BA<br>Treatment course: 8 weeks<br>Frequency: 6 times a day<br>Duration: 40 min	SLT<br>Treatment course: 8 weeks<br>Frequency: 6 times a day<br>Duration: 40 min	-	-	-	-
Yang 2011	SA<br>Treatment course: 25 days<br>Frequency: once a day<br>Duration: 30 min	BA<br>Treatment course: 25 days<br>Frequency: once a day<br>Duration: 30 min	T: 112.0±60.9<br>C: 114.0±56.1	T: 22.2±17.6<br>C: 20.5±17.7	T: 26.0±24.0<br>C: 28.0±27.5	T: 37.0±32.4<br>C: 36.4±30.9
Yang X 2015	SLT+BA+SA<br>Treatment course: NA<br>Frequency: twice a day<br>Duration: 30 min	SLT<br>Treatment course: NA<br>Frequency: once a day<br>Duration: 30 min	-	-	-	-

Supplementary Table S2. *Cont*

Study	Interventions		Baseline comprehension score	Baseline oral expression score	Baseline repetition score	Baseline denomination score
	T	C				
Yu 2018	SLT+BA<br>Treatment course: 4 weeks<br>Frequency: 6 times a week<br>Duration: 30 min	SLT<br>Treatment course: 4 weeks<br>Frequency: 6 times a week<br>Duration: 30 min	-	-	-	-
Zhang 2022	SLT+SA+TA<br>Treatment course: 4 weeks<br>Frequency: once a day<br>Duration: 90 min	SLT<br>Treatment course: 4 weeks<br>Frequency: once a day<br>Duration: 60 min	-	-	-	-
Zhang R 2020	SLT+SA<br>Treatment course: 48 days<br>Frequency: 6 times a day<br>Duration: 60 min	SLT<br>Treatment course: 48 days<br>Frequency: 6 times a day<br>Duration: 30 min	-	-	-	-
Zhang S 2010	SLT+BA+SA<br>Treatment course: 5 weeks<br>Frequency: 6 times a week<br>Duration: 60-70 min	SLT<br>Treatment course: 5 weeks<br>Frequency: 6 times a week<br>Duration: 30-40 min	-	-	-	-
Zhang Y 2012	SLT+BA+SA<br>Treatment course: 4 weeks<br>Frequency: twice a day<br>Duration: 30 min	SLT<br>Treatment course: 4 weeks<br>Frequency: twice a day<br>Duration: 20 min	-	-	-	-

Supplementary Table S2. *Cont*

Study	Interventions		Baseline comprehension score	Baseline oral expression score	Baseline repetition score	Baseline denomination score
	T	C				
Zhang YJ 2020	SLT+SA<br>Treatment course: one month<br>Frequency: once a day<br>Duration: NA	SLT<br>Treatment course: one month<br>Frequency: once a day<br>Duration: NA	-	-	-	-
Zhao 2004	SLT+BA<br>Treatment course: one month<br>Frequency: once a day<br>Duration: 30 min	SLT<br>Treatment course: one month<br>Frequency: once a day<br>Duration: 30 min	-	-	-	-
Zhao D 2021	SLT+BA<br>Treatment course: one month<br>Frequency: five times a week<br>Duration: 60 min	SLT<br>Treatment course: one month<br>Frequency: five times a week<br>Duration: 30 min	-	-	-	-
Zhou 2012	BA+SA+TA<br>Treatment course: NA<br>Frequency: once a day<br>Duration: 75 min	SLT<br>Treatment course: NA<br>Frequency: once a day<br>Duration: 45 min	T: 12.08±2.13<br>C: 10.51±2.07	T: 2.48±0.62<br>C: 1.82±0.56	T: 5.64±1.21<br>C: 4.59±1.23	T: 7.14±0.96<br>C: 6.58±0.94

Note: M, Male; F, Female; T, Treatment Group; C, Control Group; SA, scalp acupuncture; BA, body acupuncture; TA, tongue acupuncture; SLT, speech and language training

Supplementary Table S3. Risk of bias assessments for included studies

Study	Random sequence generation	Allocation concealment	Blinding of participants and personnel	Blinding of outcome assessment	Incomplete outcome data	Selective reporting	Other bias
Bao 2020	Low risk	Unclear risk	High risk	Unclear risk	Low risk	Low risk	Unclear risk
Chen 2006	Low risk	Unclear risk	Unclear risk	Unclear risk	Low risk	Low risk	Unclear risk
Chen X 2021	Low risk	Unclear risk	Unclear risk	Unclear risk	Low risk	Low risk	Unclear risk
Cheng X 2018	Unclear risk	Unclear risk	Unclear risk	Unclear risk	Low risk	Low risk	Unclear risk
Dong X 2020	Low risk	Unclear risk	High risk	Unclear risk	Low risk	Low risk	Unclear risk
Dong Y 2009	Low risk	Unclear risk	Unclear risk	Unclear risk	Low risk	Low risk	Unclear risk
Feng X 2007	Unclear risk	Unclear risk	Unclear risk	Unclear risk	Low risk	Low risk	Unclear risk
He 2021	Low risk	Unclear risk	High risk	Unclear risk	Low risk	Low risk	Unclear risk
He A 2014	Low risk	Unclear risk	High risk	Unclear risk	Low risk	High risk	Unclear risk
Hou 2018	Unclear risk	Unclear risk	High risk	Unclear risk	Low risk	Low risk	Unclear risk
Hou W 2012	Low risk	Unclear risk	Unclear risk	Unclear risk	Low risk	Low risk	Unclear risk
Huang H 2009	Unclear risk	Unclear risk	High risk	Unclear risk	Low risk	Low risk	Unclear risk
Huang S 2016	Unclear risk	Unclear risk	High risk	Unclear risk	Low risk	Low risk	Unclear risk
Jiang 2008	Low risk	Unclear risk	Unclear risk	Unclear risk	Low risk	Low risk	Unclear risk

Study	Random sequence generation	Allocation concealment	Blinding of participants and personnel	Blinding of outcome assessment	Incomplete outcome data	Selective reporting	Other bias
Li 2019	Low risk	Unclear risk	Unclear risk	Unclear risk	Low risk	Low risk	Unclear risk
Li L 2020	Low risk	Unclear risk	High risk	Unclear risk	Low risk	Low risk	Unclear risk
Li G 2006	Unclear risk	Unclear risk	Unclear risk	Unclear risk	Low risk	Low risk	Unclear risk
Li Q 2017	Low risk	Unclear risk	Unclear risk	Unclear risk	Low risk	Low risk	Unclear risk
Li S 2014	Unclear risk	Unclear risk	Unclear risk	Unclear risk	Low risk	Low risk	Unclear risk
Li X 2009	Low risk	Unclear risk	Unclear risk	Unclear risk	Low risk	Low risk	Unclear risk
Li Y 2019	Low risk	Unclear risk	High risk	Unclear risk	Low risk	Low risk	Unclear risk
Li Z 2019	Low risk	Unclear risk	Unclear risk	Unclear risk	Low risk	Low risk	Unclear risk
Li ZZ 2019	Low risk	Unclear risk	Unclear risk	Unclear risk	Low risk	Low risk	Unclear risk
Li ZP 2004	Low risk	Unclear risk	Unclear risk	Unclear risk	Low risk	Low risk	Unclear risk
Li ZP 2005	Low risk	Unclear risk	Unclear risk	Unclear risk	Low risk	Low risk	Unclear risk
Liu 2018	Unclear risk	Unclear risk	High risk	Unclear risk	Low risk	Low risk	Unclear risk
Liu 2018	Unclear risk	Unclear risk	High risk	Unclear risk	Low risk	Low risk	Unclear risk
Liu J 2018	Low risk	Unclear risk	High risk	Unclear risk	Low risk	Low risk	Unclear risk
Liu L 2006	Unclear risk	Unclear risk	Unclear risk	Unclear risk	Low risk	Low risk	Unclear risk
Liu X 2011	High risk	Unclear risk	Unclear risk	Unclear risk	Low risk	Low risk	Unclear risk

Study	Random sequence generation	Allocation concealment	Blinding of participants and personnel	Blinding of outcome assessment	Incomplete outcome data	Selective reporting	Other bias
Lu Q 2010	Low risk	Unclear risk	Unclear risk	Unclear risk	Low risk	Low risk	Unclear risk
Luo 2008	Low risk	Unclear risk	High risk	Unclear risk	Low risk	Low risk	Unclear risk
Luo 2008	Low risk	Unclear risk	High risk	Unclear risk	Low risk	Low risk	Unclear risk
Mi 2004	Low risk	Unclear risk	Unclear risk	Unclear risk	Low risk	Low risk	Unclear risk
Qin 2018	Unclear risk	Unclear risk	Unclear risk	Unclear risk	Low risk	Low risk	Unclear risk
Qiu 2020	Low risk	Unclear risk	Unclear risk	Unclear risk	Low risk	Low risk	Unclear risk
Shi 2021	Unclear risk	Unclear risk	High risk	Unclear risk	Low risk	Low risk	Unclear risk
Song 2017	Low risk	Unclear risk	Unclear risk	Unclear risk	Low risk	Low risk	Unclear risk
Song Z 2019	Low risk	Unclear risk	High risk	Unclear risk	Low risk	Low risk	Unclear risk
Tian 2012	Unclear risk	Unclear risk	Unclear risk	Unclear risk	Low risk	Low risk	Unclear risk
Tong 2017	Low risk	Unclear risk	High risk	Unclear risk	Low risk	Low risk	Unclear risk
Wang 2015	Low risk	Unclear risk	Unclear risk	Unclear risk	Low risk	Low risk	Unclear risk
Wang L 2011	Low risk	Unclear risk	High risk	Low risk	Low risk	Low risk	Unclear risk
Wang M 2016	Low risk	Unclear risk	High risk	Unclear risk	Low risk	Low risk	Unclear risk
Wang P 1999	Unclear risk	Unclear risk	Unclear risk	Unclear risk	Low risk	Low risk	Unclear risk
Wang Q	Unclear risk	Unclear risk	High risk	Unclear risk	Low risk	Low risk	Unclear risk

2018

Study	Random sequence generation	Allocation concealment	Blinding of participants and personnel	Blinding of outcome assessment	Incomplete outcome data	Selective reporting	Other bias
Wang S 2006	Low risk	Unclear risk	High risk	Unclear risk	Low risk	Low risk	Unclear risk
Wang T 2016	Low risk	Unclear risk	Unclear risk	Unclear risk	Low risk	Low risk	Unclear risk
Wang W 2009	Unclear risk	Unclear risk	Unclear risk	Unclear risk	Low risk	Low risk	Unclear risk
Wang Y 2021	Low risk	Unclear risk	Unclear risk	Unclear risk	Low risk	Low risk	Unclear risk
Wei 2021	Low risk	Unclear risk	Unclear risk	Unclear risk	Low risk	Low risk	Low risk
Wei T 2005	Unclear risk	Unclear risk	Unclear risk	Unclear risk	Low risk	Low risk	Unclear risk
Wen 2019	Unclear risk	Unclear risk	Unclear risk	Unclear risk	Low risk	Low risk	Unclear risk
Wu 2011	Unclear risk	Unclear risk	Unclear risk	Unclear risk	Low risk	Low risk	Unclear risk
Wu M 2008	High risk	Unclear risk	High risk	Unclear risk	Low risk	Low risk	Unclear risk
Xie M 2015	Unclear risk	Unclear risk	Unclear risk	Unclear risk	Low risk	Low risk	Unclear risk
Xu 2021	Unclear risk	Unclear risk	Unclear risk	Unclear risk	Low risk	Low risk	Unclear risk
Xu M 2021	Unclear risk	Unclear risk	Unclear risk	Low risk	Low risk	Low risk	Unclear risk
Yang 2011	Low risk	Unclear risk	Unclear risk	Unclear risk	Low risk	Low risk	Unclear risk
Yang X 2015	Low risk	Unclear risk	High risk	Unclear risk	Low risk	Low risk	Unclear risk
Yu 2018	Low risk	Unclear risk	High risk	Unclear risk	Low risk	Low risk	Unclear risk

Zhang 2022	Low risk	Unclear risk	Unclear risk	Unclear risk	Low risk	Low risk	Unclear risk
Study	Random sequence generation	Allocation concealment	Blinding of participants and personnel	Blinding of outcome assessment	Incomplete outcome data	Selective reporting	Other bias
Zhang R 2020	Unclear risk	Unclear risk	Unclear risk	Unclear risk	Low risk	Low risk	Unclear risk
Zhang S 2010	Unclear risk	Unclear risk	Unclear risk	Unclear risk	Low risk	Low risk	Unclear risk
Zhang Y 2012	Low risk	Unclear risk	Unclear risk	Unclear risk	Low risk	Low risk	Unclear risk
Zhang YJ 2020	Unclear risk	Unclear risk	High risk	Unclear risk	Low risk	Low risk	Unclear risk
Zhao 2004	Unclear risk	Unclear risk	Unclear risk	Unclear risk	Low risk	Low risk	Unclear risk
Zhao D 2021	Low risk	Unclear risk	High risk	Unclear risk	Low risk	Low risk	Unclear risk
Zhou 2012	High risk	Unclear risk	High risk	Unclear risk	Low risk	Low risk	Unclear risk

Supplementary Figure S1. The risk of bias evaluation

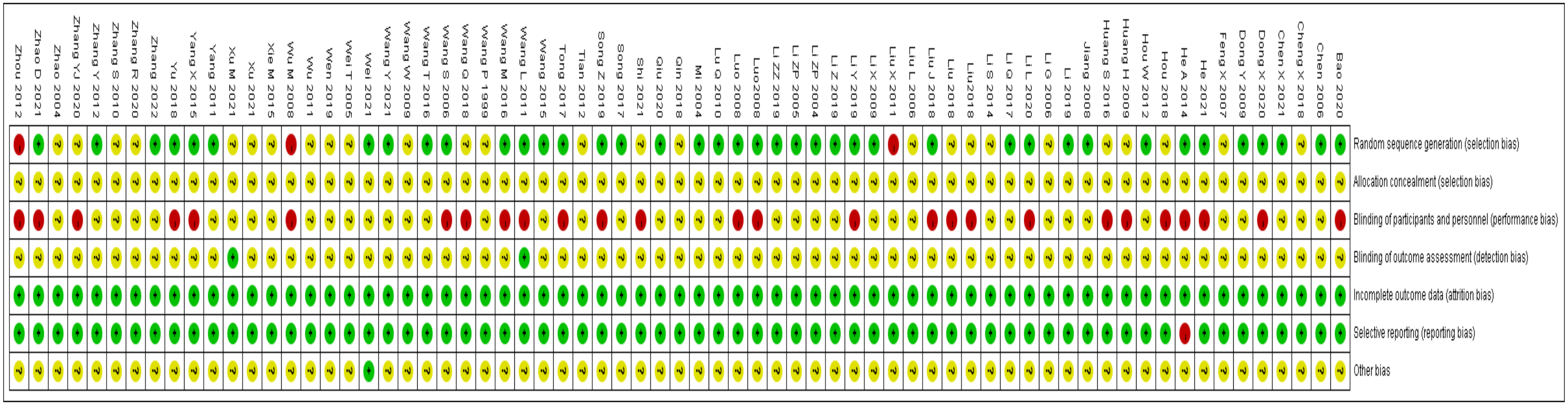

Supplementary Figure S2. Consistency assessment of network meta-analysis of total effective rate.

(A) Design inconsistency

```
Multivariate meta-analysis
Variance-covariance matrix = proportional .5*I(13)+.5*J(13,13,1)
Method = reml                                   Number of dimensions     =     13
Restricted log likelihood = -317.82131          Number of observations   =     63
------------------------------------------------------------------------------
             |      Coef.   Std. Err.      z    P>|z|     [95% Conf. Interval]
-------------+----------------------------------------------------------------
_y_B         |
       _cons |   2.087648   1.256477     1.66   0.097    -.3750012    4.550297
-------------+----------------------------------------------------------------
_y_C         |
       _cons |   .2700787   .6976747     0.39   0.699    -1.097339    1.637496
-------------+----------------------------------------------------------------
_y_D         |
       _cons |   1.079659   .3964348     2.72   0.006     .3026607    1.856657
-------------+----------------------------------------------------------------
_y_E         |
       _cons |   .5344414   .9476065     0.56   0.573    -1.322833    2.391716
-------------+----------------------------------------------------------------
_y_F         |
      des_DF |   .9766229   .9660156     1.01   0.312     -.916733    2.869979
       _cons |  -1.069684   .2688327    -3.98   0.000    -1.596587    -.542782
-------------+----------------------------------------------------------------
_y_G         |
      des_FG |  -1.192271   .8911031    -1.34   0.181    -2.938801    .5542591
       _cons |   1.548813   .8242641     1.88   0.060    -.0667147    3.164341
-------------+----------------------------------------------------------------
_y_H         |
       _cons |   .6354014   .3775453     1.68   0.092    -.1045737    1.375377
-------------+----------------------------------------------------------------
_y_I         |
       _cons |   .2887665   .6124118     0.47   0.637    -.9115386    1.489072
-------------+----------------------------------------------------------------
_y_J         |
       _cons |   .4762793   .5151479     0.92   0.355     -.533392    1.485951
-------------+----------------------------------------------------------------
_y_K         |
      des_FK |  -1.628182   1.273297    -1.28   0.201    -4.123797    .8674337
       _cons |   1.572794   1.212542     1.30   0.195    -.8037446    3.949334
-------------+----------------------------------------------------------------
_y_L         |
       _cons |   .5368939   .4351439     1.23   0.217    -.3159724     1.38976
-------------+----------------------------------------------------------------
_y_M         |
       _cons |   .0198159   .4406767     0.04   0.964    -.8438946    .8835264
-------------+----------------------------------------------------------------
_y_N         |
       _cons |   1.238172   .3266341     3.79   0.000     .5979804    1.878363
------------------------------------------------------------------------------

Estimated between-studies SDs and correlation matrix:
            SD        _y_B        _y_C        _y_D        _y_E        _y_F        _y_G
_y_B  5.689e-06          1           .           .           .           .           .
_y_C  5.689e-06         .5           1           .           .           .           .
_y_D  5.689e-06         .5          .5           1           .           .           .
_y_E  5.689e-06         .5          .5          .5           1           .           .
_y_F  5.689e-06         .5          .5          .5          .5           1           .
_y_G  5.689e-06         .5          .5          .5          .5          .5           1
_y_H  5.689e-06         .5          .5          .5          .5          .5          .5
_y_I  5.689e-06         .5          .5          .5          .5          .5          .5
_y_J  5.689e-06         .5          .5          .5          .5          .5          .5
_y_K  5.689e-06         .5          .5          .5          .5          .5          .5
_y_L  5.689e-06         .5          .5          .5          .5          .5          .5
_y_M  5.689e-06         .5          .5          .5          .5          .5          .5
_y_N  5.689e-06         .5          .5          .5          .5          .5          .5

          _y_H        _y_I        _y_J        _y_K        _y_L        _y_M        _y_N
_y_B         .           .           .           .           .           .           .
_y_C         .           .           .           .           .           .           .
_y_D         .           .           .           .           .           .           .
_y_E         .           .           .           .           .           .           .
_y_F         .           .           .           .           .           .           .
_y_G         .           .           .           .           .           .           .
_y_H         1           .           .           .           .           .           .
_y_I        .5           1           .           .           .           .           .
_y_J        .5          .5           1           .           .           .           .
_y_K        .5          .5          .5           1           .           .           .
_y_L        .5          .5          .5          .5           1           .           .
_y_M        .5          .5          .5          .5          .5           1           .
_y_N        .5          .5          .5          .5          .5          .5           1

Testing for inconsistency:
 ( 1)  [_y_F]des_DF = 0
 ( 2)  [_y_G]des_FG = 0
 ( 3)  [_y_K]des_FK = 0

           chi2(  3) =    3.92
         Prob > chi2 =    0.2698
mvmeta command stored as F9; test command stored as F8
```

(B) Side split

```
. network sidesplit all, tau

Side     Direct                Indirect              Difference                  tau
         Coef.     Std. Err.  Coef.     Std. Err.  Coef.     Std. Err.  P>|z|
A D       1.079676   .396438    .273355   .8756235   .8063207   .9611865  0.402     1.15e-0
> 9
A E       .5346768   .9476915  -.923659   .8437946   1.458336     1.2689  0.250     9.18e-0
> 7
A F      -1.070015   .2688755   .1320487  .5592825  -1.202064    .620557  0.053     2.53e-0
> 6
A G       1.548813   .8242644   .4962466  .3260444   1.052567   .8864066  0.235     3.43e-0
> 6
A N              .          .          .         .          .          .      .
> .
B E *    -1.553348   .8251933   .9957336  200.0191  -2.549082   200.0243  0.990     1.41e-0
> 7
C F *    -1.339774   .6438184  -.3490605  200.0054  -.9907139   200.0085  0.996     1.66e-0
> 7
D F      -1.172696   .8388934  -1.979017  .4692371   .8063204   .9612123  0.402     2.88e-0
> 9
E K       1.038348   .7564999  -.4198238  1.018732   1.458171   1.268908  0.250     1.91e-0
> 6
F G       1.426217   .2058684   2.479015  .8621053  -1.052797   .8863361  0.235     2.14e-1
> 0
F H *     1.705087   .2650838   3.393852  66.68839  -1.688764   66.68786  0.980     8.84e-0
> 9
F I *     1.358452   .5502519    3.04718  115.4444  -1.688728   115.4431  0.988     1.52e-0
> 9
F J *     1.545964   .4394387   3.234624  115.4442   -1.68866   115.4433  0.988     3.27e-0
> 6
F K       1.014294   .2806302   2.472542  1.237316  -1.458249   1.268726  0.250     2.05e-0
> 6
F L *     1.606578   .3421683   3.295302  89.41209  -1.688724   89.41143  0.985      .00001
> 3
F M *       1.0895   .3491775   2.778229  99.97494   -1.68873   99.97433  0.987     8.82e-0
> 9

* Warning: all the evidence about these contrasts comes from the trials which directly com
> pare them.
See help file for more information.
```

Supplementary Figure S3. Consistency assessment of network meta-analysis of the comprehension score.

(A) Design inconsistency

```
. network meta i
Command is: mvmeta _y _S  , bscovariance(exch 0.5) longparm suppress(uv mm) eq(_y_K: des_FK) vars(_y_B _y_C _y_D _y_E _y_F _y_G _y_H _y
> _I _y_J _y_K _y_L _y_M _y_N)
Note: using method reml
Note: regressing _y_B on (nothing)
Note: regressing _y_C on (nothing)
Note: regressing _y_D on (nothing)
Note: regressing _y_E on (nothing)
Note: regressing _y_F on (nothing)
Note: regressing _y_G on (nothing)
Note: regressing _y_H on (nothing)
Note: regressing _y_I on (nothing)
Note: regressing _y_J on (nothing)
Note: regressing _y_K on des_FK
Note: regressing _y_L on (nothing)
Note: regressing _y_M on (nothing)
Note: regressing _y_N on (nothing)
Note: 22 observations on 13 variables
Note: variance-covariance matrix is proportional to .5*I(13)+.5*J(13,13,1)

initial:       log likelihood = -175.24213
rescale:       log likelihood = -175.24213
rescale eq:    log likelihood = -125.45368
Iteration 0:   log likelihood = -125.45368
Iteration 1:   log likelihood =  -124.6743
Iteration 2:   log likelihood =  -124.6199
Iteration 3:   log likelihood =  -124.6197
Iteration 4:   log likelihood =  -124.6197

Multivariate meta-analysis
Variance-covariance matrix = proportional .5*I(13)+.5*J(13,13,1)
Method = reml                                 Number of dimensions    =    13
Restricted log likelihood = -124.6197         Number of observations  =    22

------------------------------------------------------------------------------
             |      Coef.   Std. Err.      z    P>|z|     [95% Conf. Interval]
-------------+----------------------------------------------------------------
_y_B         |
       _cons |   21.86634   23.26591     0.94   0.347    -23.73401    67.46668
-------------+----------------------------------------------------------------
_y_C         |
       _cons |  -22.16936   17.08792    -1.30   0.195    -55.66106    11.32235
-------------+----------------------------------------------------------------
_y_D         |
       _cons |   4.969225    9.91094     0.50   0.616    -14.45586    24.39431
-------------+----------------------------------------------------------------
_y_E         |
       _cons |    11.0641   18.80886     0.59   0.556     -25.8006    47.92879
-------------+----------------------------------------------------------------
_y_F         |
       _cons |  -23.94825   10.21614    -2.34   0.019    -43.97151   -3.924991
-------------+----------------------------------------------------------------
_y_G         |
       _cons |  -8.672997   12.29574    -0.71   0.481    -32.77221    15.42622
-------------+----------------------------------------------------------------
_y_H         |
       _cons |  -16.32001   14.14729    -1.15   0.249    -44.04818    11.40817
-------------+----------------------------------------------------------------
_y_I         |
       _cons |  -27.52533   17.08433    -1.61   0.107    -61.01001    5.959351
-------------+----------------------------------------------------------------
_y_J         |
       _cons |  -13.78533   17.16986    -0.80   0.422    -47.43764    19.86699
-------------+----------------------------------------------------------------
_y_K         |
      des_FK |  -40.23643   29.14222    -1.38   0.167    -97.35413    16.88127
       _cons |   11.44111   23.16753     0.49   0.621    -33.96643    56.84864
-------------+----------------------------------------------------------------
_y_L         |
       _cons |  -10.04702   15.61818    -0.64   0.520    -40.65809    20.56406
-------------+----------------------------------------------------------------
_y_M         |
       _cons |  -14.12498   14.12732    -1.00   0.317    -41.81402    13.56406
-------------+----------------------------------------------------------------
_y_N         |
       _cons |       1.19   14.07848     0.08   0.933    -26.40331    28.78331
------------------------------------------------------------------------------

Estimated between-studies SDs and correlation matrix:
             SD        _y_B        _y_C        _y_D        _y_E        _y_F        _y_G        _y_H        _y_I        _y_J        _y_K
_y_B  13.773283           1           .           .           .           .           .           .           .           .           .
_y_C  13.773283          .5           1           .           .           .           .           .           .           .           .
_y_D  13.773283          .5          .5           1           .           .           .           .           .           .           .
_y_E  13.773283          .5          .5          .5           1           .           .           .           .           .           .
_y_F  13.773283          .5          .5          .5          .5           1           .           .           .           .           .
_y_G  13.773283          .5          .5          .5          .5          .5           1           .           .           .           .
_y_H  13.773283          .5          .5          .5          .5          .5          .5           1           .           .           .
_y_I  13.773283          .5          .5          .5          .5          .5          .5          .5           1           .           .
_y_J  13.773283          .5          .5          .5          .5          .5          .5          .5          .5           1           .
_y_K  13.773283          .5          .5          .5          .5          .5          .5          .5          .5          .5           1
_y_L  13.773283          .5          .5          .5          .5          .5          .5          .5          .5          .5          .5
_y_M  13.773283          .5          .5          .5          .5          .5          .5          .5          .5          .5          .5
_y_N  13.773283          .5          .5          .5          .5          .5          .5          .5          .5          .5          .5

           _y_L        _y_M        _y_N
_y_B          .           .           .
_y_C          .           .           .
_y_D          .           .           .
_y_E          .           .           .
_y_F          .           .           .
_y_G          .           .           .
_y_H          .           .           .
_y_I          .           .           .
_y_J          .           .           .
_y_K          .           .           .
_y_L          1           .           .
_y_M         .5           1           .
_y_N         .5          .5           1

Testing for inconsistency:
 ( 1)  [_y_K]des_FK = 0

           chi2(  1) =    1.91
         Prob > chi2 =    0.1674
mvmeta command stored as F9; test command stored as F8
```

(B) Side split

```
. network sidesplit all, tau
```

Side	Direct Coef.	Std. Err.	Indirect Coef.	Std. Err.	Difference Coef.	Std. Err.	P>|z|	tau
A D	.	.	.	.	.	.	.	.
A E	12.3	19.50281	-27.3748	21.63832	39.6748	29.13034	0.173	13.78431
A F	-27.81606	10.55927	8.274284	19.16401	-36.09034	21.94092	0.100	13.41144
A N	.	.	.	.	.	.	.	.
B E *	-11.02	15.02928	1.590601	204.0522	-12.6106	204.6327	0.951	14.83913
C F *	-1.57	14.82219	-36.26228	202.6463	34.69228	203.189	0.864	14.81316
E K	.4797153	13.8103	-40.2749	26.06162	40.75462	29.49465	0.167	13.7864
F G *	15.06083	7.457188	54.06919	102.863	-39.00835	103.1253	0.705	14.79704
F H *	7.406587	10.60085	45.64503	144.0183	-38.23844	144.3889	0.791	14.80666
F I *	-3.8	14.81544	34.06364	202.6454	-37.86364	203.1857	0.852	14.81206
F J *	9.94	14.91399	47.80363	202.6517	-37.86363	203.1848	0.852	14.81207
F K	-5.073574	14.43644	35.71819	25.25064	-40.79176	29.07928	0.161	13.7565
F L *	13.70084	12.58703	51.93928	144.1764	-38.23844	144.3889	0.791	14.80665
F M *	9.61743	10.56077	47.85587	144.0143	-38.23844	144.389	0.791	14.80666

Supplementary Figure S4. Consistency assessment of network meta-analysis of the oral expression score.

(A) Design inconsistency

```
.  network meta i
Command is: mvmeta _y _S  , bscovariance(exch 0.5) longparm suppress(uv mm) eq(_y_J: des_FJ) vars(_y_B _y_C _y_D _y_E _y_F _y_G _y_H _y
> _I _y_J _y_K _y_L _y_M _y_N)
Note: using method reml
Note: regressing _y_B on (nothing)
Note: regressing _y_C on (nothing)
Note: regressing _y_D on (nothing)
Note: regressing _y_E on (nothing)
Note: regressing _y_F on (nothing)
Note: regressing _y_G on (nothing)
Note: regressing _y_H on (nothing)
Note: regressing _y_I on (nothing)
Note: regressing _y_J on des_FJ
Note: regressing _y_K on (nothing)
Note: regressing _y_L on (nothing)
Note: regressing _y_M on (nothing)
Note: regressing _y_N on (nothing)
Note: 21 observations on 13 variables
Note: variance-covariance matrix is proportional to .5*I(13)+.5*J(13,13,1)

initial:       log likelihood = -185.19524
rescale:       log likelihood = -185.19524
rescale eq:    log likelihood = -105.86775
Iteration 0:   log likelihood = -105.86775
Iteration 1:   log likelihood =  -105.4341
Iteration 2:   log likelihood = -105.42152
Iteration 3:   log likelihood = -105.42148
Iteration 4:   log likelihood = -105.42148

Multivariate meta-analysis
Variance-covariance matrix = proportional .5*I(13)+.5*J(13,13,1)
Method = reml                                   Number of dimensions    =     13
Restricted log likelihood = -105.42148          Number of observations  =     21
------------------------------------------------------------------------------
             |      Coef.   Std. Err.      z    P>|z|     [95% Conf. Interval]
-------------+----------------------------------------------------------------
_y_B         |
       _cons |   12.88654   13.63969     0.94   0.345    -13.84677    39.61984
-------------+----------------------------------------------------------------
_y_C         |
       _cons |  -11.26915   11.14219    -1.01   0.312    -33.10744    10.56915
-------------+----------------------------------------------------------------
_y_D         |
       _cons |   9.058867   5.325156     1.70   0.089    -1.378247    19.49598
-------------+----------------------------------------------------------------
_y_E         |
       _cons |   3.435784   9.956651     0.35   0.730    -16.07889    22.95046
-------------+----------------------------------------------------------------
_y_F         |
       _cons |  -11.97613   6.397526    -1.87   0.061    -24.51505    .5627856
-------------+----------------------------------------------------------------
_y_G         |
       _cons |   -4.80764   9.191737    -0.52   0.601    -22.82311    13.20783
-------------+----------------------------------------------------------------
_y_H         |
       _cons |  -11.52643    11.1581    -1.03   0.302    -33.39591    10.34305
-------------+----------------------------------------------------------------
_y_I         |
       _cons |  -10.92643   11.18001    -0.98   0.328    -32.83885    10.98599
-------------+----------------------------------------------------------------
_y_J         |
      des_FJ |  -21.45664   16.31699    -1.31   0.189    -53.43736    10.52409
       _cons |   10.74152   13.52941     0.79   0.427    -15.77563    37.25868
-------------+----------------------------------------------------------------
_y_K         |
       _cons |  -3.947427   9.177269    -0.43   0.667    -21.93454    14.03969
-------------+----------------------------------------------------------------
_y_L         |
       _cons |   -6.82643   11.28584    -0.60   0.545    -28.94628    15.29342
-------------+----------------------------------------------------------------
_y_M         |
       _cons |  -5.873177   9.191737    -0.64   0.523    -23.88865     12.1423
-------------+----------------------------------------------------------------
_y_N         |
       _cons |       12.7   9.449647     1.34   0.179    -5.820968    31.22097
------------------------------------------------------------------------------

Estimated between-studies SDs and correlation matrix:
             SD        _y_B        _y_C        _y_D        _y_E        _y_F        _y_G        _y_H        _y_I        _y_J        _y_K
_y_B  9.1488117           1           .           .           .           .           .           .           .           .           .
_y_C  9.1488117          .5           1           .           .           .           .           .           .           .           .
_y_D  9.1488117          .5          .5           1           .           .           .           .           .           .           .
_y_E  9.1488117          .5          .5          .5           1           .           .           .           .           .           .
_y_F  9.1488117          .5          .5          .5          .5           1           .           .           .           .           .
_y_G  9.1488117          .5          .5          .5          .5          .5           1           .           .           .           .
_y_H  9.1488117          .5          .5          .5          .5          .5          .5           1           .           .           .
_y_I  9.1488117          .5          .5          .5          .5          .5          .5          .5           1           .           .
_y_J  9.1488117          .5          .5          .5          .5          .5          .5          .5          .5           1           .
_y_K  9.1488117          .5          .5          .5          .5          .5          .5          .5          .5          .5           1
_y_L  9.1488117          .5          .5          .5          .5          .5          .5          .5          .5          .5          .5
_y_M  9.1488117          .5          .5          .5          .5          .5          .5          .5          .5          .5          .5
_y_N  9.1488117          .5          .5          .5          .5          .5          .5          .5          .5          .5          .5

           _y_L        _y_M        _y_N
_y_B          .           .           .
_y_C          .           .           .
_y_D          .           .           .
_y_E          .           .           .
_y_F          .           .           .
_y_G          .           .           .
_y_H          .           .           .
_y_I          .           .           .
_y_J          .           .           .
_y_K          .           .           .
_y_L          1           .           .
_y_M         .5           1           .
_y_N         .5          .5           1

Testing for inconsistency:
 ( 1)  [_y_J]des_FJ = 0

           chi2(  1) =    1.73
         Prob > chi2 =    0.1885
mvmeta command stored as F9; test command stored as F8

.
```

(B) Side split

```
. network sidesplit all, tau
```

Side	Direct Coef.	Direct Std. Err.	Indirect Coef.	Indirect Std. Err.	Difference Coef.	Difference Std. Err.	P>|z|	tau
A D	.	.	.	.	.	.	.	.
A E	3.6	10.05318	-17.67703	12.79894	21.27703	16.27511	0.191	9.146012
A F	-12.58273	6.500719	7.545771	13.32017	-20.1285	14.81676	0.174	9.069372
A N	.	.	.	.	.	.	.	.
B E *	-9.51	9.820077	.6947349	201.3835	-10.20473	201.6442	0.960	9.597741
C F *	-.66	9.596399	-16.71631	201.0682	16.05631	201.2972	0.936	9.595293
E J	7.319567	9.218439	-14.19066	13.54423	21.51022	16.38373	0.189	9.15296
F G *	7.137589	6.937425	24.58431	142.4536	-17.44672	142.6081	0.903	9.597004
F H *	.4	9.61478	17.78129	201.0691	-17.38129	201.297	0.931	9.595257
F I *	1	9.640193	18.38129	201.0701	-17.38129	201.2968	0.931	9.595257
F J	1.211225	6.530242	22.83342	14.942	-21.6222	16.30613	0.185	9.147432
F K *	7.978653	6.922088	25.42537	142.4524	-17.44672	142.6075	0.903	9.597004
F L *	5.1	9.762735	22.48129	201.078	-17.38129	201.2988	0.931	9.595257
F M *	6.076454	6.936439	23.52317	142.4529	-17.44672	142.6074	0.903	9.597004

* Warning: all the evidence about these contrasts comes from the trials which directly compare them.
See help file for more information.

Supplementary Figure S5. Consistency assessment of network meta-analysis of the repetition score.

(A) Design inconsistency

```
. network meta i,force
Warning: inconsistency model requested, but there is no source of inconsistency
Command is: mvmeta _y _S  , bscovariance(exch 0.5) longparm suppress(uv mm)  vars(_y_B _y_C _y_D _y_E _y_F _y_G _y_H _y_I _y_J _y_K _y_
> L _y_M)
Note: using method reml
Note: using variables _y_B _y_C _y_D _y_E _y_F _y_G _y_H _y_I _y_J _y_K _y_L _y_M
Note: 22 observations on 12 variables
Note: variance-covariance matrix is proportional to .5*I(12)+.5*J(12,12,1)

initial:       log likelihood = -133.13844
rescale:       log likelihood = -133.13844
rescale eq:    log likelihood = -119.78085
Iteration 0:   log likelihood = -119.78085
Iteration 1:   log likelihood = -118.43412
Iteration 2:   log likelihood = -118.41295
Iteration 3:   log likelihood = -118.41288
Iteration 4:   log likelihood = -118.41288

Multivariate meta-analysis
Variance-covariance matrix = proportional .5*I(12)+.5*J(12,12,1)
Method = reml                                 Number of dimensions    =    12
Restricted log likelihood = -118.41288        Number of observations  =    22

------------------------------------------------------------------------------
             |      Coef.   Std. Err.      z    P>|z|     [95% Conf. Interval]
-------------+----------------------------------------------------------------
_y_B         |
       _cons |   12.89487   10.17193     1.27   0.205    -7.041745    32.83148
-------------+----------------------------------------------------------------
_y_C         |
       _cons |  -11.17635   7.106912    -1.57   0.116    -25.10564    2.752939
-------------+----------------------------------------------------------------
_y_D         |
       _cons |   7.424173    3.29611     2.25   0.024     .9639149    13.88443
-------------+----------------------------------------------------------------
_y_E         |
       _cons |   1.811135   8.279279     0.22   0.827    -14.41595    18.03822
-------------+----------------------------------------------------------------
_y_F         |
       _cons |  -12.24301    4.56166    -2.68   0.007     -21.1837   -3.302324
-------------+----------------------------------------------------------------
_y_G         |
       _cons |  -1.394082   5.398967    -0.26   0.796    -11.97586    9.187698
-------------+----------------------------------------------------------------
_y_H         |
       _cons |   -11.2749   7.264613    -1.55   0.121    -25.51328    2.963481
-------------+----------------------------------------------------------------
_y_I         |
       _cons |  -4.076808   6.045231    -0.67   0.500    -15.92524    7.771627
-------------+----------------------------------------------------------------
_y_J         |
       _cons |  -10.19899   6.432506    -1.59   0.113    -22.80647    2.408493
-------------+----------------------------------------------------------------
_y_K         |
       _cons |   -.646166   6.087602    -0.11   0.915    -12.57765    11.28531
-------------+----------------------------------------------------------------
_y_L         |
       _cons |  -5.733842   6.081619    -0.94   0.346     -17.6536    6.185912
-------------+----------------------------------------------------------------
_y_M         |
       _cons |      8.941   5.952693     1.50   0.133    -2.726063    20.60806
------------------------------------------------------------------------------

Estimated between-studies SDs and correlation matrix:
             SD       _y_B       _y_C       _y_D       _y_E       _y_F       _y_G       _y_H       _y_I       _y_J       _y_K
_y_B  5.4477966          1          .          .          .          .          .          .          .          .          .
_y_C  5.4477966         .5          1          .          .          .          .          .          .          .          .
_y_D  5.4477966         .5         .5          1          .          .          .          .          .          .          .
_y_E  5.4477966         .5         .5         .5          1          .          .          .          .          .          .
_y_F  5.4477966         .5         .5         .5         .5          1          .          .          .          .          .
_y_G  5.4477966         .5         .5         .5         .5         .5          1          .          .          .          .
_y_H  5.4477966         .5         .5         .5         .5         .5         .5          1          .          .          .
_y_I  5.4477966         .5         .5         .5         .5         .5         .5         .5          1          .          .
_y_J  5.4477966         .5         .5         .5         .5         .5         .5         .5         .5          1          .
_y_K  5.4477966         .5         .5         .5         .5         .5         .5         .5         .5         .5          1
_y_L  5.4477966         .5         .5         .5         .5         .5         .5         .5         .5         .5         .5
_y_M  5.4477966         .5         .5         .5         .5         .5         .5         .5         .5         .5         .5

           _y_L       _y_M
_y_B          .          .
_y_C          .          .
_y_D          .          .
_y_E          .          .
_y_F          .          .
_y_G          .          .
_y_H          .          .
_y_I          .          .
_y_J          .          .
_y_K          .          .
_y_L          1          .
_y_M         .5          1

Testing for inconsistency:
 ( 1)  - [_y_F]_cons + [_y_L]_cons = 0

           chi2(  1) =    2.66
         Prob > chi2 =    0.1027

mvmeta command stored as F9; test command stored as F8
```

(B) Side split

```
. network sidesplit all, tau
```

Side	Direct Coef.	Direct Std. Err.	Indirect Coef.	Indirect Std. Err.	Difference Coef.	Difference Std. Err.	P>|z|	tau
A D	.	.	.	.	.	.	.	.
A E *	1.9	8.309305	-11.11	100.1763	13.01	100.5204	0.897	5.450283
A F *	-12.59985	4.600461	-.0750002	26.7656	-12.52485	27.1581	0.645	5.438156
A M	.	.	.	.	.	.	.	.
B E *	-11.11	5.938693	14.91	200.9251	-26.02	201.0406	0.897	5.450283
C F *	-1.05	5.456588	-23.48061	200.4299	22.43061	200.5046	0.911	5.448312
F G *	10.83071	2.873847	35.51752	100.5317	-24.68681	100.5656	0.806	5.445609
F H *	.95	5.660151	25.48498	200.4364	-24.53498	200.5046	0.903	5.448182
F I *	8.148091	3.97342	32.73351	141.879	-24.58542	141.928	0.862	5.44731
F J *	2.025832	4.496299	26.61126	141.8877	-24.58542	141.9238	0.862	5.44731
F K *	11.57879	4.070435	36.16421	141.8811	-24.58542	141.9255	0.862	5.44731
F L *	6.490995	3.989496	31.07642	141.8741	-24.58542	141.9248	0.862	5.44731

* Warning: all the evidence about these contrasts comes from the trials which directly compare them.
See help file for more information.

Supplementary Figure S6. Consistency assessment of network meta-analysis of the denomination score.

(A) Design inconsistency

```
. network meta i,force
Warning: inconsistency model requested, but there is no source of inconsistency
Command is: mvmeta _y _S  , bscovariance(exch 0.5) longparm suppress(uv mm)  vars(_y_B _y_C _y_D _y_E _y_F _y_G _y_H _y_I _y_J _y_K _y_
> L _y_M)
Note: using method reml
Note: using variables _y_B _y_C _y_D _y_E _y_F _y_G _y_H _y_I _y_J _y_K _y_L _y_M
Note: 21 observations on 12 variables
Note: variance-covariance matrix is proportional to .5*I(12)+.5*J(12,12,1)

initial:       log likelihood = -133.15587
rescale:       log likelihood = -133.15587
rescale eq:    log likelihood = -117.04799
Iteration 0:   log likelihood = -117.04799
Iteration 1:   log likelihood = -115.92591
Iteration 2:   log likelihood = -115.85359
Iteration 3:   log likelihood =  -115.8531
Iteration 4:   log likelihood =  -115.8531

Multivariate meta-analysis
Variance-covariance matrix = proportional .5*I(12)+.5*J(12,12,1)
Method = reml                                  Number of dimensions    =     12
Restricted log likelihood = -115.8531          Number of observations  =     21
```

	Coef.	Std. Err.	z	P>|z|	[95% Conf.	Interval]
_y_B						
_cons	11.85383	10.70813	1.11	0.268	-9.133725	32.84138
_y_C						
_cons	-15.58017	7.822605	-1.99	0.046	-30.9122	-.2481463
_y_D						
_cons	5.145267	3.475142	1.48	0.139	-1.665887	11.95642
_y_E						
_cons	1.812174	8.583494	0.21	0.833	-15.01117	18.63551
_y_F						
_cons	-16.16738	5.131967	-3.15	0.002	-26.22585	-6.108912
_y_G						
_cons	-6.32276	5.9847	-1.06	0.291	-18.05256	5.407037
_y_H						
_cons	-8.519283	7.86125	-1.08	0.278	-23.92705	6.888485
_y_I						
_cons	-15.93928	7.821727	-2.04	0.042	-31.26959	-.6089801
_y_J						
_cons	-11.09928	7.943215	-1.40	0.162	-26.6677	4.469133
_y_K						
_cons	-16.96865	7.004958	-2.42	0.015	-30.69812	-3.239185
_y_L						
_cons	-7.12981	6.673833	-1.07	0.285	-20.21028	5.950663
_y_M						
_cons	-9.795988	6.83008	-1.43	0.152	-23.1827	3.590723

```
Estimated between-studies SDs and correlation matrix:
             SD       _y_B       _y_C       _y_D       _y_E       _y_F       _y_G       _y_H       _y_I       _y_J       _y_K
_y_B  5.9060448          1          .          .          .          .          .          .          .          .          .
_y_C  5.9060448         .5          1          .          .          .          .          .          .          .          .
_y_D  5.9060448         .5         .5          1          .          .          .          .          .          .          .
_y_E  5.9060448         .5         .5         .5          1          .          .          .          .          .          .
_y_F  5.9060448         .5         .5         .5         .5          1          .          .          .          .          .
_y_G  5.9060448         .5         .5         .5         .5         .5          1          .          .          .          .
_y_H  5.9060448         .5         .5         .5         .5         .5         .5          1          .          .          .
_y_I  5.9060448         .5         .5         .5         .5         .5         .5         .5          1          .          .
_y_J  5.9060448         .5         .5         .5         .5         .5         .5         .5         .5          1          .
_y_K  5.9060448         .5         .5         .5         .5         .5         .5         .5         .5         .5          1
_y_L  5.9060448         .5         .5         .5         .5         .5         .5         .5         .5         .5         .5
_y_M  5.9060448         .5         .5         .5         .5         .5         .5         .5         .5         .5         .5

           _y_L       _y_M
_y_B          .          .
_y_C          .          .
_y_D          .          .
_y_E          .          .
_y_F          .          .
_y_G          .          .
_y_H          .          .
_y_I          .          .
_y_J          .          .
_y_K          .          .
_y_L          1          .
_y_M         .5          1

Testing for inconsistency:
 ( 1)  - [_y_F]_cons + [_y_M]_cons = 0

           chi2(  1) =    2.07
         Prob > chi2 =    0.1506

mvmeta command stored as F9; test command stored as F8
```

(B) Side split

```
. network sidesplit all, tau
```

Side	Direct Coef.	Direct Std. Err.	Indirect Coef.	Indirect Std. Err.	Difference Coef.	Difference Std. Err.	P>|z|	tau
A D	.	.	.	.	.	.	.	.
A E *	1.9	8.617721	-10.07	100.2071	11.97	100.577	0.905	5.909836
A F *	-16.76374	5.153272	-.0400001	26.7722	-16.72374	27.26368	0.540	5.872216
B E *	-10.07	6.437388	13.87	201.0187	-23.94	201.1541	0.905	5.909836
C F *	-.56	5.909951	-31.85291	200.5252	31.29291	200.6126	0.876	5.905314
F G *	9.816182	3.055067	42.48504	100.6573	-32.66886	100.699	0.746	5.896996
F H *	7.62	5.960712	40.03586	200.5287	-32.41586	200.614	0.872	5.905183
F I *	.2	5.908488	32.61586	200.5237	-32.41586	200.6105	0.872	5.905183
F J *	5.04	6.068401	37.45586	200.5274	-32.41586	200.6094	0.872	5.905183
F K *	-.8295375	4.749459	31.67038	141.9941	-32.49992	142.0388	0.819	5.90242
F L *	9.00956	4.284307	41.50948	141.9814	-32.49992	142.0387	0.819	5.90242
F M *	6.34273	4.430681	38.84265	141.9817	-32.49992	142.0402	0.819	5.90242

* Warning: all the evidence about these contrasts comes from the trials which directly compare them.
See help file for more information.
